# Intracellular amyloid formation in muscle cells of Aβ-transgenic *Caenorhabditis elegans*: determinants and physiological role in copper detoxification

**DOI:** 10.1186/1750-1326-4-2

**Published:** 2009-01-06

**Authors:** Alicia N Minniti, Daniela L Rebolledo, Paula M Grez, Ricardo Fadic, Rebeca Aldunate, Irene Volitakis, Robert A Cherny, Carlos Opazo, Colin Masters, Ashley I Bush, Nibaldo C Inestrosa

**Affiliations:** 1 Centro de Regulación Celular y Patología "Joaquín V. Luco" (CRCP), MIFAB, Centro de Envejecimiento y Regeneración (CARE), Facultad de Ciencias Biológicas, Pontificia Universidad Católica de Chile, Alameda 340, 8331010 Santiago, Chile; 2 Departamento de Neurología, Facultad de Medicina, Pontificia Universidad Católica de Chile, Santiago, Chile; 3 Oxidation Disorders Laboratory, Mental Health Research Institute of Victoria and Department of Pathology, University of Melbourne, Parkville, Victoria 3052, Australia; 4 Laboratorio de Neurobiometales, Departamento de Fisiología, Facultad de Ciencias Biológicas, Universidad de Concepción, Chile; 5 Department of Psychiatry, Harvard Medical School, Massachusetts General Hospital, Charlestown, Massachusetts, USA

## Abstract

**Background:**

The amyloid β-peptide is a ubiquitous peptide, which is prone to aggregate forming soluble toxic oligomers and insoluble less-toxic aggregates. The intrinsic and external/environmental factors that determine Aβ aggregation *in vivo *are poorly understood, as well as the cellular meaning of this process itself. Genetic data as well as cell biological and biochemical evidence strongly support the hypothesis that Aβ is a major player in the onset and development of Alzheimer's disease. In addition, it is also known that Aβ is involved in Inclusion Body Myositis, a common myopathy of the elderly in which the peptide accumulates intracellularly.

**Results:**

In the present work, we found that intracellular Aβ aggregation in muscle cells of *Caenorhabditis elegans *overexpressing Aβ peptide is affected by two single amino acid substitutions, E22G (Arctic) and V18A (NIC). Both variations show decrease intracellular amyloidogenesis compared to wild type Aβ. We show that intracellular amyloid aggregation of wild type Aβ is accelerated by Cu^2+ ^and diminished by copper chelators. Moreover, we demonstrate through toxicity and behavioral assays that Aβ-transgenic worms display a higher tolerance to Cu^2+ ^toxic effects and that this resistance may be linked to the formation of amyloid aggregates.

**Conclusion:**

Our data show that intracellular Aβ amyloid aggregates may trap excess of free Cu^2+ ^buffering its cytotoxic effects and that accelerated intracellular Aβ aggregation may be part of a cell protective mechanism.

## Background

Aberrant protein aggregation is a common feature of late-onset amyloidogenic diseases, such as Alzheimer's disease (AD) and Inclusion Body Myositis (IBM) [1]. A peptide of 39–43 mer derived from the amyloid precursor protein (APP), the amyloid β-peptide (Aβ), is the main constituent of senile plaques (SPs) in AD [2], and it is also one of the hallmarks of IBM, a common myopathy characterized by the presence of intracellular amyloid aggregates in skeletal muscle cells [1,3]. IBM patients show progressive muscle weakness, compromised muscle innervation [4] and muscle fibre degeneration [5]. Besides the presence of senile plaque-like inclusions, most molecules known to be involved in IBM are also present in AD [6].

Dominant mutations in the APP gene are associated with rare cases of familial AD, in which brain and vascular amyloid deposits are formed earlier than in sporadic cases [7]. One type of familial AD is linked to a point mutation in the Aβ peptide (E22G, Arctic) that accelerates aggregation of Aβ into protofibrils and fibrils *in vitro *[8-12]. Some transgenic mice carrying the Arctic mutation show increased plaque formation but normal learning and memory compared to strains carrying the wild type Aβ [13]. Opposite to the Arctic variant, *in vitro *studies demonstrated that the NIC mutation (V18A), which is predicted to favour the α helix conformation over the β sheet conformation, shows decreased aggregation [14].

Although APP and Aβ have been implicated in several processes *in vitro *and *in vivo*, such as neuronal development and cell survival, the *in vivo *functions of APP and Aβ remain unclear [7,15,16]. Moreover, the inactivation of the complete APP gene family has shown that APP is necessary for neurodevelopment and cell adhesion [17]. In addition, neither the *in vivo *mechanisms of amyloid formation nor the factors required for this process have been properly established. It is also unknown if the formation of insoluble amyloid aggregates is instrumental for the onset of these amyloidogenic diseases, or if the formation of amyloid structures is the end product of the cell protective machinery. SPs are structured as metal-enriched aggregates that accumulate Cu^2+^, Fe^3+^, and Zn^2+ ^[18-20]. In agreement with this, the aggregation state of Aβ-peptide is increased by Cu^2+ ^or Zn^2+ ^[21] and reduced by metal chelators such as clioquinol [22-24]. However, the role of copper as deleterious or beneficial in the context of amyloidogenic diseases is very controversial. For instance, in a transgenic APP23 mouse model, supplementation of the diet with bioavailable copper restored normal levels of SOD-1 activity and decreased the production of soluble Aβ [25]. From these studies, the structural plasticity of the SP is becoming evident in agreement with previous structural data [26]. In light of this evidence we postulate that the formation of the SP might be triggered by fluctuations on the levels of transition metals, and could be a part of a protective homeostatic mechanism gone off-course. However, it is unknown if these metals are accumulated in the intracellular amyloid aggregates observed in IBM and if the aggregation state of Aβ is modulated by transition metals in this intracellular muscle environment. Interestingly, IBM patients do not develop dementia and AD patients do not have the muscle weakness characteristic of IBM, which indicates that these diseases may be triggered by independent mechanisms [1].

*Caenorhabditis elegans *has an APP homologue but this protein lacks a region equivalent to the Aβ-peptide [27]. However, several studies carried out in Aβ overexpressing *C. elegans *[6] have established that the formation of amyloid deposits [28] could induce oxidative stress [29], stress response [30,31] and up- or down-regulation of different genes [32]. However, the toxic Aβ-species responsible for these effects detected in Aβ-transgenic worms have not been thoroughly identified, but they are likely to be a specific type of Aβ oligomers rather than mature Aβ amyloid aggregates [33-35]. It is important to mention that the paralysis phenotype is only observed in Aβ expressing *C. elegans *in a dominant *rol-6 *background (strains carrying a mutation in a collagen gene) [32]. In fact, the great majority of IBM patients show muscle weakness that translates in a lower quality of life but not paralysis or decreased longevity [36].

In the present work, we evaluated intrinsic and extrinsic factors on the aggregation of intracellular Aβ peptides constitutively overexpressed in muscle cells of *C. elegans*. Our results indicate that intracellular Aβ aggregation is selectively affected by single amino acid substitution, which is in agreement with previously published *in vitro *data [14]. Under our experimental conditions, we find that the NIC mutation (V18A) did form fewer Thioflavine-S (ThS)-positive aggregates compared to Aβ wt. Neither non-aggregating Aβ peptide nor aggregating Aβ peptide induced strong paralysis phenotypes in these animals in a wild type background. However, Aβ wild type affected the worms' motility as they aged mimicking the muscle weakness observed in IBM patients. Therefore, we asked the question whether CuCl_2 _could modulate the aggregation of intracellular Aβ-peptide in muscle cells, as it is apparent with the extracellular Aβ-peptide in transgenic vertebrate models for AD [22,37]. We found that Cu^2+ ^increases the number of intracellular ThS-positive aggregates present in this model. In contrast, the presence of the Cu-chelators histidine and clioquinol in the growing medium decreases the formation of amyloid deposits. Surprisingly, the Aβ-expressing animals, in spite of producing a great number of amyloid aggregates in the presence of Cu^2+^, exhibited increased tolerance to the cytotoxic effects of CuCl_2 _compared to control animals. Therefore, the present evidence suggests, for the first time, that intracellular amyloid deposits are dynamically structured by the presence of metals in muscle cells, and that the formation of intracellular Aβ aggregates could be a part of the homeostatic mechanism responsible for protecting cells against abrupt changes in Cu^2+ ^levels.

## Results

### The NIC Aβ_1–42 _variant displays a low rate formation of intracellular amyloid deposits in *C. elegans *muscle cells

**Figure 1:**
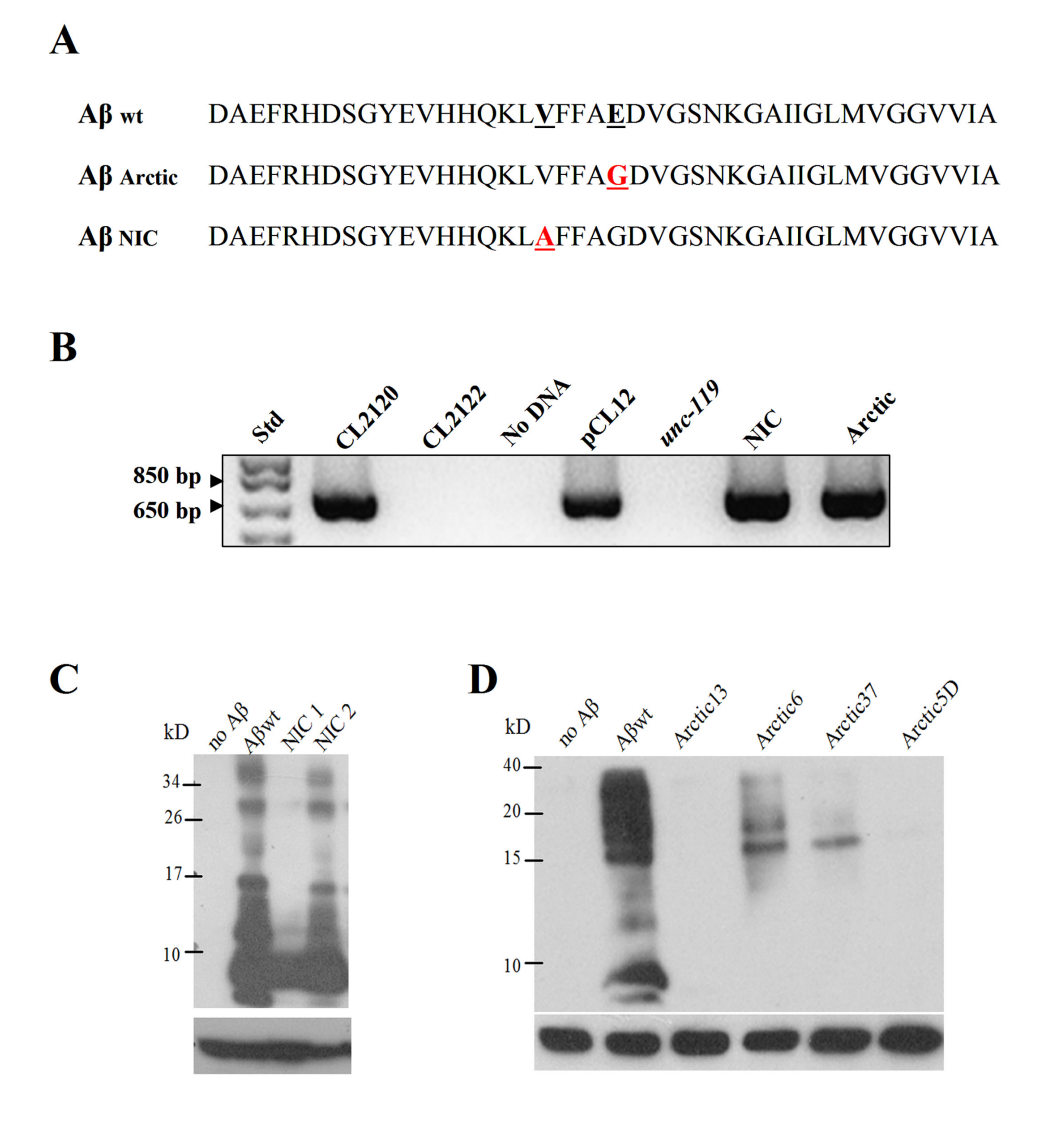
**Transgenic *C. elegans *strains express the Aβ wild type and its variants Arctic and NIC**. **A**. Amino acidic sequences of the Aβ_1–42 _peptide (wild type) and its variants Arctic and NIC expressed in the transgenic *C. elegans *strains used in this study. **B**. PCR results show the presence of the DNAs that encode the different peptides in the transgenic strains. CL2120 is the strain that express Aβ wt and CL2122 is the control strain that do not express the peptide. NIC corresponds to transgenic line NIC2 and Arctic corresponds to transgenic line Arctic 6. *unc-119 *is the strain used to construct the transgenic lines carrying the NIC and Arctic variants. **C**. Western blot analysis using the anti-Aβ antibody 6E10 showing the expression of Aβ in the CL2120 (wild type Aβ) strain and in two NIC transgenic lines (NIC 1 and NIC 2). The lane marked as "no Aβ " correspond to strain CL2122. **D**. Western blot analysis using the same antibody as in C showing the expression of Aβ in strain CL2120 (wild type Aβ) and in four Arctic transgenic lines (Arctic 13, Arctic 6, Arctic 37, Arctic 5D). The lane marked as "no Aβ " correspond to strain CL2122. α-Tubulin was used as loading control in C and D.

In order to investigate the key intrinsic determinants of the human Aβ sequence for Aβ intracellular amyloid aggregation, we constructed transgenic *C. elegans *strains that express two different variants, Arctic (E22G) and NIC (V18A) (Fig. 1A, B, C and 1D), in muscle cells as described in the Methods section. Interestingly, despite having generated several transgenic lines that expressed the Arctic variant, all of them showed levels of expression far lower than those expressing the wild type or the NIC variant (Fig. 1C and 1D). One possibility is that the overexpression of the Arctic variant in *C. elegans *muscle cells is too harmful and therefore the only surviving transgenic lines were those with low Aβ Arctic expression. For our experiments we used the strain NIC2, with Aβ load similar to that of the Aβ wt expressing strain, and Arctic 6.

**Figure 2:**
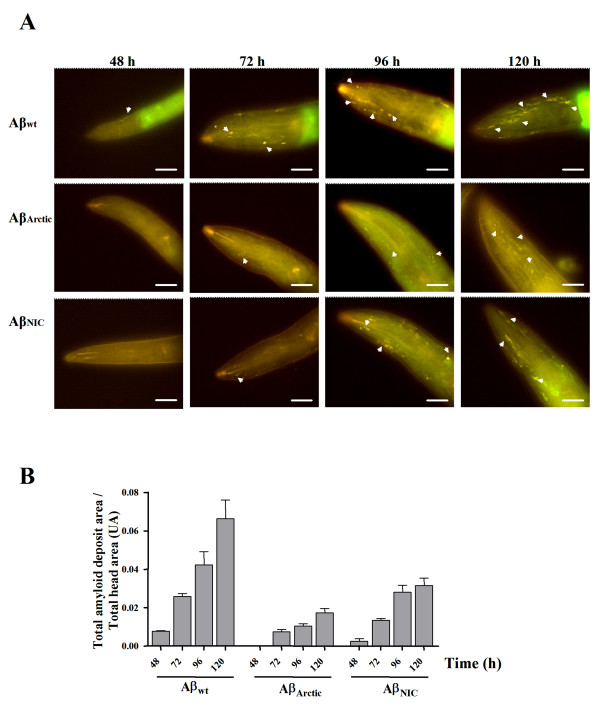
**Amyloid formation in transgenic *C. elegans *expressing different Aβ variants**. **A**. Images show ThS stained *C. elegans *that express the Aβ_1–42 _wt or its variants Arctic and NIC. The visualization of the amyloid deposits (arrow heads) in these strains is shown at 48 h (larval stage L4), 72 h (1 day old adult), 96 h (2 day old adult) and 120 h (3 day old adult) of development. The images are representative of 4 experiments where 40 worms were analyzed for each time point. Bar 20 μm. **B**. Quantification of amyloid deposit area in ThS stained transgenic *C. elegans *expressing the Aβ variants mentioned above. The quantification of the amyloid deposit area was also done at 48, 72, 96 and 120 h of development in the head of at least 40 worms for each time point, from the tip of the heat to the end of the pharynx (see Fig. 4A).

**Figure 3:**
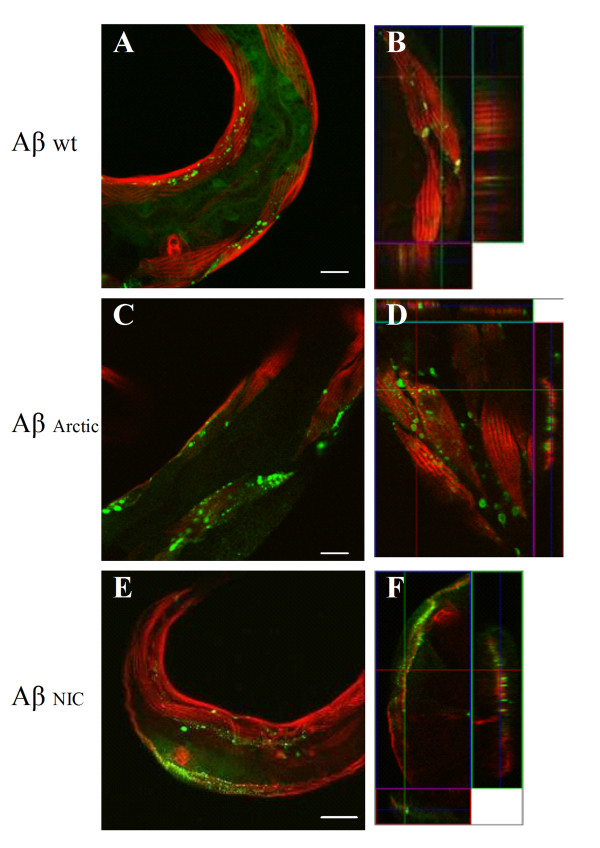
**Transgenic *C. elegans *strain expressing the Arctic Aβ peptide shows high levels of non-amyloidogenic aggregates**. Panels **A**, **C **and **E **show confocal images of 120 h old worms that express the Aβwt, the Arctic and the NIC variants, respectively, stained with anti-Aβ antibodies (green) and phaloidin (red) to show the actin filaments in body wall muscle cells. Panels **B **(Aβwt), **D **(Aβ Arctic) and **F **(Aβ NIC) show the orthogonal view of the same animals shown in A, C and E, to establish the location of the Aβ aggregates in relation to the actin filaments. Bar 20 μm.

We then analyzed the degree of amyloidosis at different times during the animal's life to establish the pattern of intracellular aggregation. Fig. 2(A, B) shows the time course of amyloid formation as visualized by ThS staining. The animals were processed and stained at 48, 72, 96 and 120 h after being synchronized at the embryo stage. All transgenic strains show an increment in the total amyloid deposit area as they age (Fig. 2B). However, the animals expressing the Aβ variants show fewer amyloid aggregates than those that express the Aβwt at all ages. The worms expressing the NIC variant start showing amyloid deposits by 48 h but to a lesser extent than those expressing the Aβwt. Over the next time points (up to 120 h) the formation of amyloid deposits increased in these animals, but to a much lesser extent than in those expressing the Aβwt (Fig. 2A, B) as anticipated from previous *in vitro *experiments [14]. We were not able to detect any ThS positive spots until 72 h in the animals that express the Arctic variant, which suggests a delay in the formation of mature amyloid deposits as expected. However, in the next time points, the degree of amyloid formation in these transgenic animals was very poor, even lower than those expressing the NIC variant (Fig. 2A, B). We expect this low level of amyloid formation of the Arctic variant is due to the poor expression of the transgene compared with the strains carrying the wild type and NIC variants (Fig. 1C, D). Fig. 3 shows the expression of the three Aβ variants in the transgenic *C. elegans *lines by immunofluorescence (120 h). They all show expression of the Aβ peptide (green) in the muscle cells identified by phalloidin staining of actin filaments (red). As shown in the orthogonal analyses (Fig. 3B, D, F) the Aβ concentrated in aggregates is mainly located between the actin filaments, which are well observed in *C. elegans *overexpressing the Aβwt variant (Fig. 3A, B). Even though the Arctic variant formed very few and small amyloid deposits (probably as a consequence of low expression of the transgene (Fig. 1D)), the peptide accumulates in what appears to be fairly big amorphous aggregates, which were Aβ-immunoreactive (Fig. 3C, D). We do not observe clear muscle degeneration in any of the transgenic strains since actin fibers look fairly intact (Fig. 3).

### Cu^2+ ^modulates intracellular Aβ amyloidosis in transgenic *C. elegans*

**Figure 4:**
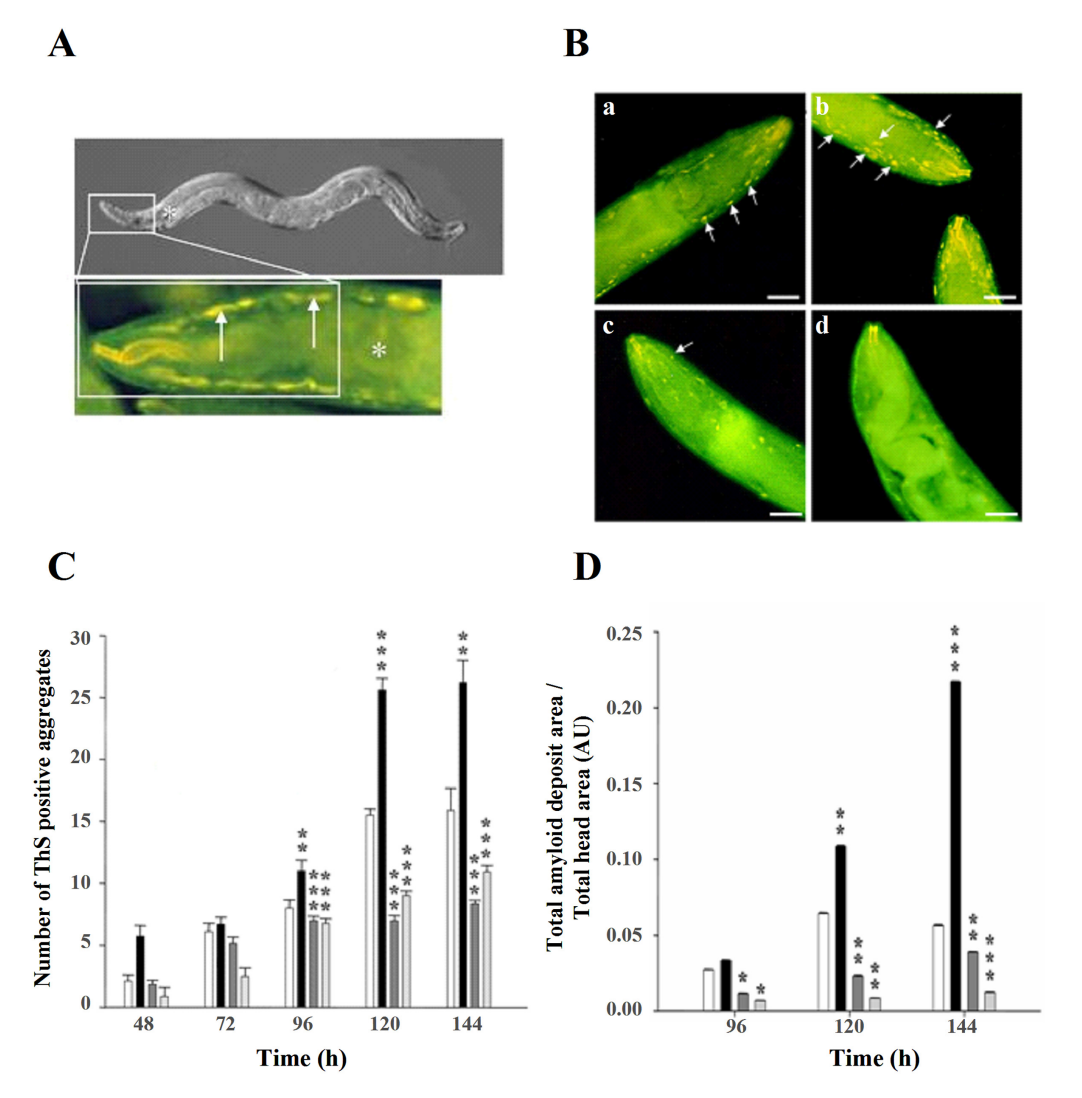
**Cu^2+ ^and Cu-chelators modulate the aggregation state of intracellular Aβwt in transgenic *C. elegans***. **A. **The white rectangle on the DIC image of a *C. elegans* hermaphrodite (top picture) indicates the region of the worm where all quantifications were done (the same region, stained with ThS in seen below). * Indicates the position of the pharyngeal bulb. **B. **Morphology of ThS-positive amyloid deposits formed in the presence of Cu^2+^ and Cu-chelators. Images correspond to *C. elegans* grown on regular NGM agar plates (a), treated with 150 μM CuCl_2_ (b), 150 μM histidine (c) or 150 μM clioquinol (d) for 144 h. The amyloid deposits were stained with ThS and photographed under an epifluorescence microscope. Arrows indicate ThS positive amyloid aggregates. The images are representative of 4 experiments where 40 worms were analyzed for each treatment. Bar 20 μm. **C.** The graph represents the number of aggregates formed anterior to the pharyngeal bulb at different times (48–144 h) under control conditions (white bars) and when the animals were treated with CuCl_2_ (150 μM, black bars), histidine (150 μM, dark grey bars), or clioquinol (150 μM, pale gray bars). Bars indicate standard error, **p < 0.01; ***p < 0.001. **D.** The graph shows the digital quantification (Sigma Scan Pro 5) of the ThS-positive amyloid deposits present anterior to the pharyngeal bulb. The graph represents the total area of aggregates formed anterior to the pharyngeal bulb at different times (96–144 h) under control conditions (white bars) and when the animals were cultured in the presence of CuCl_2_ (150 μM, black bars), histidine (150 μM, dark grey bars), or clioquinol (150 μM, pale gray bars). Bars indicate standard error, *p < 0.05; **p < 0.01; ***p < 0.001. The p values indicate differences between a given treatment and the control**.**.

To evaluate if the aggregation state of Aβ expressed in the muscle cells of the worms could be altered by environmental factors, we decided to expose the worms to copper, mainly because there is a large body of evidence indicating that the aggregation of Aβ in the brain can be modulated by transition metals [25,38,39]. We performed experiments with copper using the strain expressing the human wild type Aβ peptide, since it is the most commonly found in amyloidogenic diseases, the best characterized, and it behaves as expected in our previous studies. We first analyzed the formation of amyloid aggregates by staining synchronized populations of animals cultured on Cu^2+^, histidine and clioquinol supplemented medium every 24 h. Time zero was established as the time the eggs were harvested and seeded onto agar plates supplemented with the CuCl_2_, histidine or clioquinol. The CuCl_2 _concentrations used in the experiments were determined by preliminary studies of viability, fertility and Cu^2+ ^incorporation in the wild type (N2) strain and analysis of stress induction in the *hsp-16*:GFP transgenic strain CL2070 [31,40]. Based on these studies we decided to use a concentration of 150 μM CuCl_2_, because it did not affect the survival of the worms. Cu-chelators were also used at a concentration of 150 μM. As previously reported, ThS reactive deposits were not observed in very young animals although their presence has been established even in embryos by staining with the more sensitive Congo-red derived dye X-34 [41]. We started seeing ThS positive deposits 48 h after the eggs where seeded on the plates, when most of the animals were L4 larvae (data not shown). Before this time, it was not possible to perform a quantitative analysis between the different treatments. The number of ThS positive deposits was quantified anterior to the pharyngeal bulb in the cephalic region of the worms (Fig. 4A), as described in the Methods section. Up to 72 h of treatment, the differences in the number of amyloid deposits between the animals exposed to CuCl_2_, histidine or clioquinol did not appear significantly different from controls (Fig 4C). After 96 h of treatment, the formation of ThS-positive deposit was more abundant in the worms grown in CuCl_2 _than in the worms cultured in the control medium (Fig 4B a–b, C). At this time, the worms cultured in the agar supplemented with Cu-chelators developed scarce amyloid deposits, significantly less than the number of deposits formed in the worms grown in the control medium (Fig. 4B a–d; C). The same pattern was observed at 144 h of treatment (Fig. 4C). We confirmed this quantification by measuring the total aggregate area in the worm's cephalic region at 96, 120 and 144 h (Fig. 4D). As expected, the CuCl_2 _treatment increased the total deposit area, while histidine and clioquinol reduced it significantly. Moreover, at this time, the few aggregates formed in the presence of histidine and clioquinol seemed to have a more diffuse morphology than the ThS-amyloid aggregates formed in the presence of CuCl_2 _and controls (Fig. 4Bc and 4d). In addition, the treatment with clioquinol often produced a virtual absence of discernible aggregates (Fig 4Bd).

### Cu^2+ ^tolerance in Aβ- transgenic *C. elegans*

**Figure 5:**
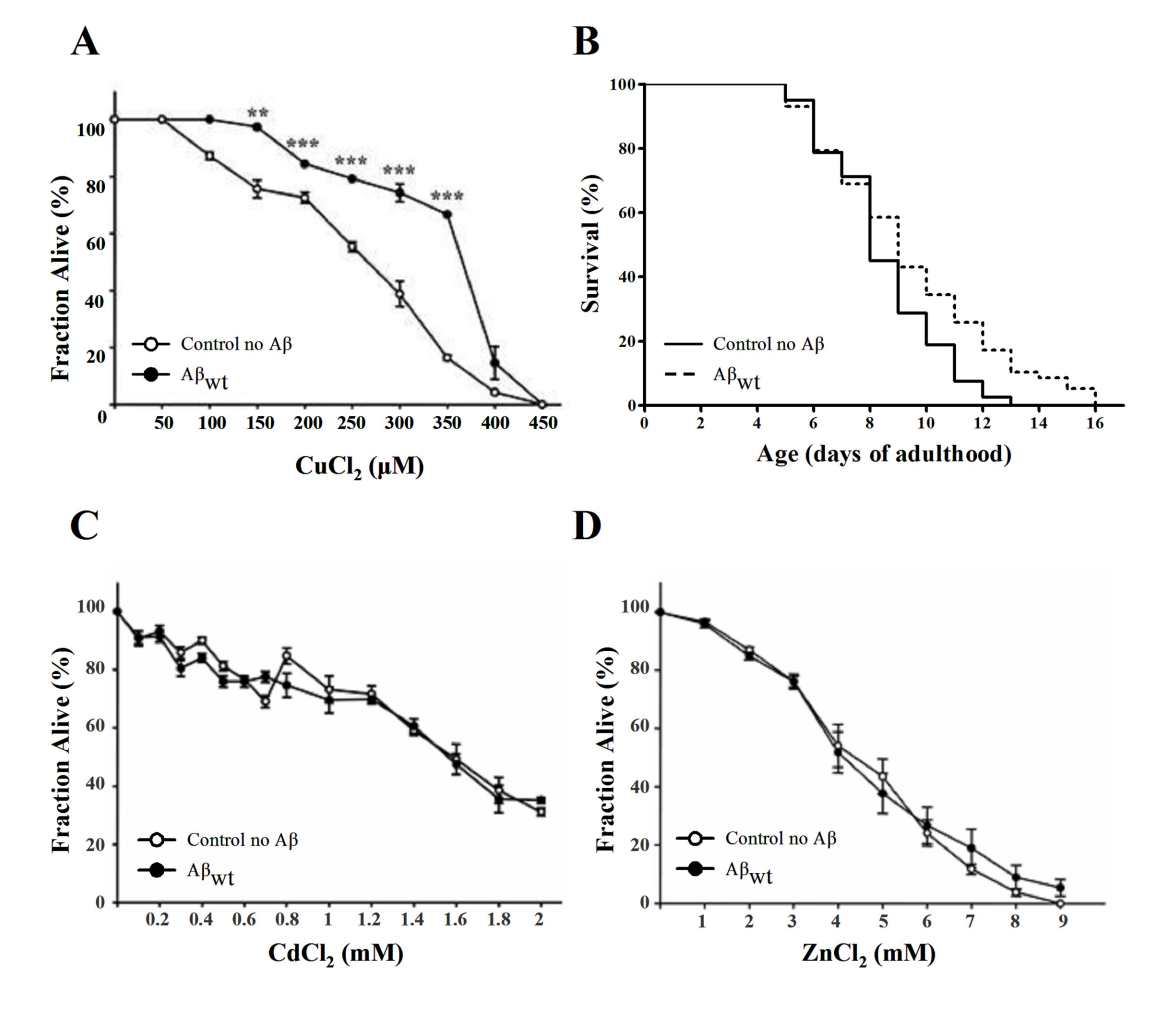
***C. elegans *that express Aβ are resistant to Cu^2+ ^toxicity**. **A**. Control (open circles) and Aβ wt expressing animals (black circles) were exposed to a range of CuCl_2 _(**A**), CdCl_2 _(**C**), and ZnCl_2 _(**D**) concentrations in liquid K medium for 24 h. The number of worms that remained alive was determined under a stereoscope by visual inspection. The experiments were performed in triplicate. Data are means ± S.E. **p < 0.01; ***p < 0.001. **B**. Survival curves of control worms and Aβ worms cultured on 150 μM CuCl_2_. Comparison of survival curves using the Log-rank (Mantel-Cox) test yielded a p value of 0.0066 (n = 138).

Aβ has a strong affinity for Cu^2+ ^[42], therefore, we set up to test whether there was a differential tolerance to the toxic effects of CuCl_2 _between the Aβ expressing worms (CL2120) and the controls (CL2122). We reasoned that if Cu^2+ ^becomes bound to Aβ, there would be less free Cu^2+ ^to cause cell damage. To test this hypothesis we performed toxicity assays in which we exposed 96 h old Aβ-expressing worms and their controls to a range of copper concentrations ranging from zero (no copper added to the medium) to 450 μM in liquid medium during 24 h. In this way we were able to define survival curves for both populations and found a significant shift to the right for the Aβ transgenic animals (Fig. 5A). The lethal concentration at which half of the population dies (LC_50_) was significantly higher for the animals expressing the Aβ peptide (366 ± 2 μM) than the controls (268 ± 8 μM). These results show that the Aβ transgenic worms are more resistant than controls to high Cu^2+ ^concentrations suggesting that there might be less metal available to produce cell damage in this strain. We confirmed that the animals exposed to high concentrations of CuCl_2 _(150 μM) during 96 and 120 h incorporated more Cu^2+ ^(≈10 μg/g) than the animals grown on regular medium (≈ 0.1 μg/g) (Table 1). Moreover, Cu^2+ ^supplementation did not induce changes in Zn^2+^, Fe^3+^, Mn^2+ ^or Al^3+ ^levels (data not shown). We did not observe any significant difference in constitutive Cu (or Zn^2+^, Fe^3+^, Mn^2+ ^and Al^3+^) levels between the Aβ-transgenic *C. elegans *and their controls, indicating that the presence of Aβ does not affect Cu^2+ ^absorption from the agar medium. Therefore, the aggregation of intracellular Aβ was mediated by direct reaction with elevated Cu^2+^. In fact, the Aβ-transgenic animals and their control counterparts exposed to CuCl_2_, incorporated about 100 times more Cu^2+ ^than the animals grown under normal culture conditions. However, we did not observe any statistically significant difference in the amount of incorporated Cu^2+ ^between the Aβ-transgenic *C. elegans *and their controls under either condition (no CuCl_2 _added and CuCl_2 _supplementation) (Table 1), indicating that the presence of Aβ is not affecting Cu^2+ ^transport, and suggesting that the effect observed on the experiments could be explained principally by the interaction between Cu^2+ ^and the Aβ present in the muscle cells of the nematode. These results show that the Aβ transgenic worms are more resistant than controls to high Cu^2+ ^concentrations.

**Table 1:** Copper incorporation into Aβ expressing *C. elegans *and controls

**Age [h]**	**Strain**	**Cu^2+ ^Content** **[μg/g protein]**	
		No copper added	CuCl_2 _150 μM

**96**	**No Aβ**	0.350 ± 0.458	8.831± 1.077
	
	**Aβ**_1–42_	0.169 ± 0.142	12.109 ± 2.523

**120**	**No Aβ**	0.128 ± 0.110	9.931 ± 0.930
	
	**Aβ**_1–42_	0.068 ± 0.015	9.224 ± 1.989

Quantification of Cu^2+ ^incorporated in control (CL2122 = no Aβ expression) and Aβ-transgenic (CL2120 = Aβ_1–42_) *C. elegans*. Worms were grown in the presence of CuCl_2 _(150 μM) or under control conditions (no copper added) for 96 and 120 h. The Cu^2+ ^content (μg/g) was determined by ICP-MS. Data are means ± S.E.

In order to confirm that the protective effect of Aβ expression is limited to copper toxicity we performed CdCl_2 _and ZnCl_2 _toxicity assays. Both metals are known to be toxic and to elicit a stress response in *C. elegans *[43-45]. Furthermore, zinc is known to be involved in the metallobiology of Alzheimer's disease [46]. We found that the survival curves (Fig. 5C, D) are almost identical between Aβ expressing worms and controls in both cases. The differences in LC_50 _for cadmium (CL2120: 1.55 ± 0.02 mM; CL2122: 1.59 ± 0.03 mM) and zinc (CL2120: 4.9 ± 0.8 mM; CL2122: 4.8 ± 0.4 mM) are not statistically significant between the strains. These results exclude the possibility that Aβ overexpression may be providing a more general protective effect, at least to metal toxicity.

Finally, to correlate amyloid aggregate load with resistance to copper toxicity, we performed lifespan experiments to determine if Aβ expressing worms show longer lifespan than controls under 150 μM copper. We found that Aβ worms show a small but significant increase in median and total lifespan (Fig. 5B) demonstrating the protective effect of Aβ when the worms were chronically exposed to copper. When cultured on regular agar, the Aβ expressing worms and the control animals show a similar lifespan (Additional file 1).

### Aβ- transgenic *C. elegans *are protected from Cu^2+ ^toxicity in behavioral assays

**Figure 6:**
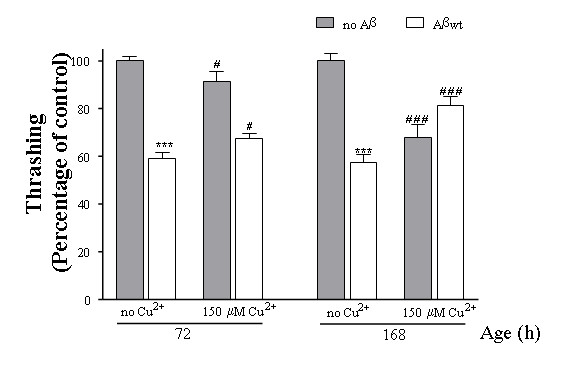
**Aβ-transgenic *C. elegans *are protected from *Cu*^2+ ^toxicity in motility assays**. The graph represents the results of the thrashing assays (movement in liquid medium) performed on control and Aβ expressing worms after being cultured on regular agar or in copper supplemented agar for 72 or 168 h stating at the embryo stage. Statistical analysis: t-test to compare control worms (grey bars) with Aβ expressing worms (white bars) cultured on regular agar plates (no Cu^2+^) during 72 and 168 h. *** p < 0.001. t-test to compare control worms cultured on copper supplemented agar with control worms cultured on regular agar during 72 and 168 h. # p < 0.005, ### p < 0.001; and to compare Aβ worms cultured on copper supplemented agar (150 μM) with Aβ worms cultured on regular agar during 72 and 168 h. # p < 0.005, ### p < 0.001. Bars indicate standard error. See representative videos in Additional files 2 and 3.

To further corroborate the protective effect of Aβ expression to copper toxicity we performed thrashing assays [47]. These assays measure motor deficits in liquid medium and have been used extensively in studies of mutants that affect the function and/or structure of the *C. elegans *neuromuscular junction [47-49]. We first evaluated the motility of Aβ expressing worms and controls that had been cultured in regular agar plates for 72 and 120 h (from the embryo stage). We found that the transgenic Aβ animals move 40% slower than controls (Fig. 6), indicating that Aβ is indeed affecting the worms' behaviour, even though this is not evident when they crawl on agar plates. Then, we evaluated the motility of Aβ expressing animals and their controls after being cultured on agar medium supplemented with 150 μM CuCl_2 _during the same periods of time (72 and 168 h). We found that while the control animals show decreased movement at both time points after being cultured in copper rich agar plates, the Aβ expressing worms are not affected under the same conditions and they even show significant improvement (Fig. 6 and Additional files 2 and 3).

## Discussion

Most of the studies performed to understand intracellular Aβ aggregation and amyloid formation have been done using *in vitro *systems. Therefore, neither the *in vivo *mechanisms of amyloid formation nor the factors required for this process have been established [15]. In the present work, we evaluated intrinsic and extrinsic factors in the aggregation of Aβ peptides constitutively overexpressed in muscle cells of *Caenorhabditis elegans*. Under our experimental conditions, we found that Aβ peptide carrying the Arctic (E22G) or the NIC mutation (V18A) did form few ThS-positive aggregates compared to Aβwt. The results obtained with the NIC (V18A) variant are in agreement with previously published *in vitro *data [14]. This mutation is expected to favor the α helix over the β sheet conformation, which would cause a decrease in amyloidosis [14]. However, the results obtained with the Arctic mutation (E22G) are at variance with the literature [50]. The initial delay in the formation of aggregates is in agreement with the kinetics of aggregation of the Arctic variant *in vitro *in which there is a lag in the formation of amyloid in favor of the development of protofibrils, when compared with the Aβwt [12]. However, the poor amyloidogenesis in later time points (even lower than the NIC variant) is probably due to low transgene expression since the Arctic variant is known to be highly amyloidogenic as established in studies of transgenic mice expressing this human variant in the nervous system [13,50]. Our inability to produce transgenic lines that constitutively express high levels of Aβ Arctic could be related to the deleterious effect of high levels of this variant during embryonic stages that may have preclude us from obtaining such strains. On the other hand, the immunofluorescence experiments show that much of the Aβ Arctic is aggregated in what appears to be amorphous deposits. Perhaps, there are muscle expressed factors that prevent the formation of mature amyloid deposits of the Aβ Arctic variant.

In our model, Aβwt peptide expression induced a clear decrease in the worms' motility (Fig. 6), which could be compared to the progressive muscle weakness present in IBM patients. The lack of muscle degeneration observed in the Aβwt expressing animals suggests that the Aβ peptide might not be sufficient to induce a stronger myopathy by itself in the muscle cells of worms, and that extra factors might be needed to fully replicate IBM symptoms in *C. elegans*. The situation could be very similar to that seen in triple transgenic mice models for AD, where mice need to overexpress Aβ together with Tau and presenilin (all mutant variants) to replicate the brain human features characteristic of the pathology [51]. Therefore, Aβ apparently needs to act with other factor/s present in the muscle to fully induce IBM symptoms in worm muscle [1].

We also showed that Cu^2+ ^and the Cu^2+ ^chelators histidine and clioquinol can modulate the formation of intracellular amyloid aggregates in *C. elegans *muscle cells. Copper ionophores like clioquinol and PBT2 show benefits in transgenic mouse models and phase 2 clinical trials of AD [22,24,52]. Despite the differences in cellular location, intracellular Aβ aggregation in *C. elegans *seems to be enhanced by Cu^2+ ^and diminished by histidine and clioquinol in accordance with previous *in vitro *and *in vivo *reports [22,23]. The increased amyloidosis could also be the result of higher Aβ expression brought about by Cu exposure and lower Aβ expression caused by Cu-chelator treatment. This situation is not likely to be the case because the constitutive *unc-54 *promoter is driving Aβ expression. Moreover, it has been shown that in addition to aggregated Aβ-peptide there is an important pool of soluble peptide in worms expressing Aβ [30]. Therefore, increased peptide synthesis is not necessary for an eventual increment in Aβ for interaction and subsequent aggregation in the presence of Cu^2+^. We did, however, evaluate Aβ expression in worms subjected to the different treatments by semi-quantitative RT-PCR and found that there were no significant differences in its expression (data not shown). Therefore, these results support a pivotal role of Cu^2+ ^in the aggregation state of intracellular Aβ.

In the present study, the animals were treated continuously with CuCl_2_, histidine or clioquinol since they were embryos; at this stage there is no Aβ aggregation that can be observed by ThS staining although some aggregates have been detected with the X-34 dye [41]. Therefore, we have yet to establish whether treating animals with histidine or clioquinol later in life, once a significant number of deposits have been formed, can eliminate or reduce the number and/or size of the aggregates. Although we have shown an effect of Cu^2+^, histidine and clioquinol on the formation of intracellular amyloid deposits in muscle cells by histochemical staining with ThS, we have not yet resolved the question of the relative concentrations of soluble versus insoluble aggregated Aβ and their changes upon treatment with Cu-chelators. It would be very informative to clarify this point by double staining with ThS or X-34 to visualize the deposits, and Aβ antibodies that recognize both soluble and insoluble forms of the amyloid peptide. This experiment would clarify whether Cu^2+ ^and Cu-chelators influence the equilibrium between both Aβ forms.

The differences observed among individuals exposed to the same treatments could be due to the developmental stage at which the animals start their exposure to the different agents. Even though we start the treatments on embryos, there is some difference in the actual developmental stage of the embryo (data not shown). Although this is a possibility, we do not think it is the case, since we have observed similar differences in animals cultured on standard medium. It is unlikely that the differences observed between same age individuals are due to genetic background. Self-fertilizing reproduction in *C. elegans *precludes much genetic variation in the strains. The strains used in our studies are transgenic strains in which several copies of Aβ had been integrated into the genome. However, integration of a transgene into the genome does not always prevent mosaicism and/or differences in expression between individuals [53].

Another caveat is that we do not know if there is enrichment of metals (Cu, Zn and others) in the intracellular amyloid deposits of *C. elegans *muscle cells as is the case in the extracellular amyloid aggregates found in AD affected neuronal tissue [20]. The determination of metal levels in the muscle aggregates would be an important step in drawing a firmer analogy between AD extracellular brain aggregates and intracellular muscle deposits.

Perhaps the most striking finding is that the Aβ-expressing animals, in spite of producing great number of amyloid aggregates in the presence of Cu^2+^, exhibited increased tolerance to the cytotoxic effects of CuCl_2 _compared to control animals and suggests that there might be less metal available to produce cell damage in this strain. The results from the behavioural experiments together with the results obtained in the toxicity assays offer strong evidence that Aβ may be providing protection to Cu^2+ ^toxicity. Interestingly, the Aβ expressing worms that were exposed to CuCl_2 _move better than those that were not cultured in the presence of this transition metal, suggesting that perhaps the increase in amyloid deposits in these treated worms is limiting the amount of the more toxic oligomeric species [33-35]. Our findings connect with those of other authors showing that increased Aβ deposition associates with decrease levels of neuronal oxidative damage in Down Syndrome and AD [54-57]. It has also been suggested that the chelation of redox-active copper and iron may be the most important mechanism by which Aβ exerts its protective function [58], and that at moderate copper concentrations, Aβ acts as an antioxidant to prevent Cu^2+ ^catalyzed oxidation of biomolecules [59]. The levels of intracellular copper must be strictly regulated to prevent aberrant reactive oxygen species generation resulting in neuronal and muscle damage. In fact, intracellular free copper has been estimated to be almost nil [60], and is under the control of a robust metalloprotein array that rapidly and efficiently buffers free copper bioavailability. However, this protein array is clearly altered in age-related diseases such as Alzheimer's disease [61], which may explain why copper is accumulated in amyloid plaques of people suffering of this disease [20]. Moreover, a more recent report described that total brain copper levels increase in aged mouse [62]. Considering that age is a risk factor for AD neuropathology, brain copper rise in elderly humans may be one of the neurochemical factors relevant in the onset of AD. Moreover, cellular acidosis could facilitate the release of copper from metal binding proteins [63]. Interestingly, brain acidosis is observed in AD [64], which may facilitate the release of copper from copper-binding proteins increasing the formation of Aβ-Cu aggregates. This may be also part of the mechanism that increases copper bioavailability in IBM.

Therefore, the present evidence suggests that intracellular amyloid deposits are dynamically structured by metals, and that Aβ may be a component of the system that specifically controls copper homeostasis at the cellular level [65-67].

Much research has been done on the possible role of copper and other metals such as zinc and iron on the development of AD, but so far no consensus has been reached in terms of the specific involvement of copper in this pathology, and conflicting reports point either to a deleterious role of copper [68] or a beneficial one [25]. Moreover, there is practically no data available about the possible role of metal homeostasis on other disorders characterized by amyloidosis such as sporadic IBM.

## Conclusion

In this study we show in a *C. elegans *model that the intracellular senile plaque-like structures formed in muscle cells and characteristic of IBM can be modulated by copper and copper chelators. The accelerated Aβ aggregation in the presence of copper seems to be linked to a higher tolerance to the cytotoxic effects of this metal. Our results suggest that the formation of intracellular Aβ aggregates could be part of a mechanism that defends the cell against sudden changes in copper concentration.

## Methods

### Solutions

Distilled H_2_O (dH_2_O) used in buffer preparations was submitted to resin treatment in order to eliminate organic compounds, cations and anions. After treatment, the resultant dH_2_O had a resistance value of 18 MΩ/cm.

### Nematode propagation and strains

Transgenic strains CL2120 and CL2122 were a gift of Dr. Christopher Link and were described earlier [28]. Briefly, CL2120 expresses the Aβ1–42 peptide under the control of the *unc-54 *(myosin heavy chain) promoter that drives expression to the body wall muscle cells. These animals form intracellular amyloid deposits constitutively in their muscle cells [69]. CL2122 has the same vector as CL2120, minus Aβ1–42, incorporated into the genome and therefore it does not form amyloid aggregates. Both strains have *mtl-2*/GFP as marker gene and therefore they express GFP in the intestinal cells. The worms were cultured on regular 35, 60 or 100 mm culture plates with NGM agar seeded with the bacterial strain OP50 [70]. The agar and the bacterial cultures were supplemented with histidine (Sigma), clioquinol (Sigma) and CuCl_2 _(Sigma) at a concentration of 150 μM. All strains were maintained at 20°C. Worm cultures were synchronized by the hypochlorite method [71] and the resulting eggs were seeded onto the previously described agar plates. The animals were collected every 24 h by washing the plates with M9 buffer [71]. The worms were precipitated in a clinical centrifuge and the M9 removed. The nematodes were then stained as described below.

### Aβ mutagenesis

Construction of the transgenic strains expressing the Aβ variants, Arctic (E22G) and NIC (V18A) (Fig. 1A), were obtained by site directed mutagenesis on the pCL12 plasmid (kindly provided by Dr. Christopher Link) that carries the human wt Aβ1–42 peptide (with a signal peptide in the N-terminus) under the control of the *unc-54 *promoter that drives expression to the body wall muscle cells [28]. The NIC mutation (V18A) induces a significant increment in the α-helical content of Aβ and dramatically diminishes fibrillogenesis *in vitro *[14]. The primers used to generate the Arctic (5'TGGTGTTCTTTGCAGGAGATGTGGGTTCAAA3' and its complementary anti-sense primer) and NIC (5'ATCATCAAAAATTGGCGTTCTTTGCAGAAGA3' and its complementary anti-sense primer) mutations were obtained commercially (Invitrogen). We obtained plasmids pPG110 and pPG120 carrying the Arctic and the NIC variant respectively as established by sequencing the DNA inserts. The transgenic lines were created using microparticle bombardment of the plasmid of interest and the selectable co-transformation marker *unc-119 *(pJN254) into *unc-119 (e2498) *mutants, as described earlier except that we used tungsten particles instead of gold particles [72]. The presence of each transgene was determined by PCR (primer sense 5'TGACCGTGGAAGATTA 3' located in the *unc-54 *promoter and primer antisense 5'GTCCAATGATTGCACCT 3' between bases 1208 and 1224 of the pCL12 plasmid and its derivatives pPG110 and pPG120 (Fig. 1B). The expression of the transgenes was established by western blot analysis (Fig. 1C and 1D) and immunofluorescence (6E10 antibody, Sigma) (Fig. 3). We obtained 4 transgenic lines that express the NIC variant and 10 expressing the Arctic variant. For Western analysis we choose lines in which the transgene was incorporated into the genome and therefore transferred to 100% of the progeny. For the analysis of intracellular aggregation (ThS and immunofluorescence) we choose lines with equivalent Aβ expression (NIC2). This was not possible for the Arctic variant since it we could not obtain strains expressing this variant at high levels, but we nevertheless analysed strain Arctic 6.

### Western blot analysis

Worms were collected form the plates with M9 buffer, transferred to tubes, centrifuged and washed two times to eliminate bacteria. The final worm pellet was resuspended in 100 μl lysis buffer (50 mM HEPES pH = 7.5; 6 mM MgCl_2_; 1 mM EDTA; 75 mM sucrose; 25 mM benzamidine; 1 mM DTT; 1% Triton X-100) and frozen at 80°C. The samples were sonicated 2 times on ice for 15 seconds and centrifuged 5 minutes at 10,000 rpm to eliminate the cuticles. The supernatant was transferred to a new tube and total protein content was quantified. Then, a volume corresponding to 50 μg protein was precipitated with acetone. The pellet was resuspended in loading buffer (50 mM Tris-HCl pH6.8; 100 mM DTT; 5% SDS; 10 % glycerol; bromophenol blue; 5% β-mercaptoethanol) and boiled for 3 minutes (adapted from Wu et al. 2006). The samples were subjected to electrophoresis on Tris-Tricine polyacrilamide gels at constant voltage (100 V) at 4°C. The proteins were then transferred to PVDF membranes for 2 hours at 4°C (300 mA). The membranes were boiled in PBS for 3 minutes before blocking with a solution of PBT plus 5% milk for one hour at room temperature. We used the anti-Aβ monoclonal antibody 6E10 (Chemicon) at 1:1,000 dilution or anti-α-tubulin monoclonal antibody (Sigma) at 1:5,000 dilution overnight at 4°C. We used goat anti-mouse HRP as secondary antibody. The densitometry analysis was performed with Matrix software.

### Immunofluorescence

We used the monoclonal anti-Aβ antibody 6E10 (Sigma) and as secondary antibody an anti-mouse Alexa Fluor 488 (Molecular Probes) pre-adsorbed with fixed worms. The fixation, permeabilization, and staining of the specimens were performed as described before [73]. Some samples were also stained with rhodamine-phalloidin (1:4,000) for the visualization of actin filaments in muscle cells.

### ThS fluorescence staining

ThS staining was performed as described before [28] with the modification that it was done in whole *C. elegans *populations instead of on individual worms. Briefly, the worms washed from the plates were submitted to a brief centrifugation at 3,000 rpm in microfuge tubes and fixed by adding 1 ml 4% paraformaldehyde in PBS pH 7.4 for 24 h at 4°C. The next day, the fixative was removed, replaced by 0.5 ml permeabilization solution (1% Triton X-100, 5% β-mercaptoethanol, 125 mM Tris, pH 7.4), and incubated at 37°C for 24 h. After removing the permeabilization solution the animals were washed in PBS-T (Triton X-100 0.1%) pH 7.4, stained in 0.125% ThS in 50% ethanol for 2 min and distained for another 2 min in 50% ethanol. The 50% ethanol was removed; the worm pellet was resuspended in a drop of PBS and a small aliquot observed under a Zeiss Axioplan microscope. If the background of the staining was too high a second wash with 50% ethanol was performed. The animals were finally mounted on a drop of mounting medium (DAKO), covered with a cover slip and sealed. The samples were coded, and the counting and analysis was performed double blind. The samples were then observed and photographed with a Zeiss Axioplan microscope equipped with epifluorescence or analysed in a Zeiss LSM 2000 MT confocal microscope. To quantify the amyloid aggregates, the ThS stained deposits present anterior to the pharyngeal bulb were counted in a minimum of 40 worms in three independent experiments.

### Image quantification and statistical analysis

Digital quantification of areas positive for ThS fluorescence were measured with the Sigma Scan Pro 5 or ImageJ. The analysis revealed the total average area of the deposits. The total area of deposits was divided by the total head area to standardize it to the worm size. Data from the image analysis microscopy were exported to a Sigma Plot file or GraphPad Prism 4 for statistical analysis. Results were expressed as mean ± standard error. Statistical significance was determined by one-way analysis of variance (ANOVA); p < 0.05 was regarded as statistically significant.

### Metal Quantification

*C. elegans *worms were grown during 96 or 120 h on regular agar plates or agar plates supplemented with 150 μM CuCl_2_. The animals were washed off the plates with dH_2_O, placed on microfuge tubes and subjected to three washes with dH_2_O, they were then heated 2 times at 100°C for 15 min. All samples were stored at 4°C. To avoid possible metal contaminations, the tubes used to collect the samples were previously treated with 0.5% HNO_3 _for 24 h, washed with dH_2_O 3 times and let dry at 20°C. The samples were digested in 200 μl of 12N HNO_3 _(BDH, ARISTAR) overnight, the next day 200 μl of 1% HNO_3 _were added. Samples were further diluted (1:10) in 1% HNO_3 _for analysis by inductive coupled plasma mass spectrometry (ICP-MS). ICP-MS was performed using an UltraMass 700 (Varian Inc., Australia). Tissue samples were prepared in triplicate for all treatments and Cu^2+ ^values for each sample was determined in triplicate. Cu^2+ ^values are mean μg/g weight of each *C. elegans *sample.

### Cu^2+ ^toxicity assays

Age-synchronous *C. elegans *populations were prepared by transferring eggs to a freshly seeded NGM plate. After 96 h, the worms were transferred to K medium (53 mM NaCl, 32 mM KCl) supplemented with different concentrations of CuCl_2 _(up to 450 μM) on 24 well microtiter plates (15–20 worms/well) for 24 h at 20°C [44]. The number of dead worms was determined by the absence of touch-provoked movement when prodded with a platinum wire. Each independent experiment of a total of three was performed in triplicate.

### Lifespan assays

Lifespan analysis was conducted at 20°C as described previously [74]. For the lifespan assays, 5–10 adult animals were allowed to lay eggs overnight on regular NGM plates seeded with OP50 bacteria. Parents were removed the next day and eggs were allowed to develop at 20°C. L4 larvae were transferred to new regular NGM plates or to 150 μM CuCl_2 _supplemented plates. This time point was used as day 0 to account for differences in developmental time between strains. The worms were transferred to new plates every day during the first 4–5 days to avoid the mixing of generations. The number of dead individuals was verified every two days. A worm was considered dead when it did not respond when prodded two times with a platinum wire pick. Worms that died with larvae inside (eggs hatched before being laid) or those in which the intestine extruded from the vulva were removed from the sample. Lifespan curves were generated and analyzed with GraphPad PRISM^® ^version 5.0. Log-rank (Mantel-Cox) test was used for statistical analysis of survival curves.

### Thrashing assays

Individual animals of the same age were placed on an 80 μl drop of M9 buffer. After a 2-minute recovery period the worms were recorded for 1.5 minutes and the thrashes counted. A thrash is defined as a change in the direction of bending at the mid body [47]. For the copper treatment experiments, the worms to be assayed were exposed to copper from the embryo (egg) stage in agar plates until the thrashing experiments were performed at 72 h (1 day-old adults), and 168 h later (5 day-old adults).

## Competing interests

AIB is a shareholder; consultant and Scientific Advisory Board member of Prana Biotechnology Ltd. CM is a director of Prana Biotechnology Ltd., Chairperson of its Scientific Advisory Committee and stockholder. IV and RC declare competing financial interests associated to Prana Biotechnology LTD.

## Authors' contributions

ANM participated in the design and coordination of the study, carried out the Aβ aggregation experiments, the toxicity assays, and drafted the manuscript, DLR performed the behavioural studies, the quantification of amyloid deposits and the statistical analysis, PMG carried out the *in vitro *mutagenesis and constructed the transgenic strains, RF contributed with the medical aspects of the study, RA performed the lifespan experiments and analysis, IV and RAC performed the metal quantifications, CO participated in the design and coordination of the study, CM participated in the design of the study, AIB participated in the design and coordination of the study and helped draft the manuscript, NCI conceived the study, participated in its design and coordination of the manuscript. All authors read and approved the final manuscript.

## Supplementary Material

Additional file 1 **Aβ transgenic *C. elegans *and controls have similar life spans.** Survival curves of control worms and Aβ worms cultured on regular agar medium. Comparison of survival curves using the Log-rank (Mantel-Cox) test yielded a p = 0.1. Survival curves are not different. Click here for file

Additional file 2 **Aβ transgenic *C. elegans *are protected from Cu^2+ ^toxicity in motility assays.** Representative video of thrashing assays (movement in liquid medium) performed on control worms of 168 h cultured on 150 μM copper supplemented agar. Click here for file

Additional file 3 **Aβ transgenic *C. elegans *are protected from Cu^2+ ^toxicity in motility assays.** Representative video of thrashing assays (movement in liquid medium) performed on control worms of 168 h cultured on 150 μM copper supplemented agar. Click here for file
