# Supplementary figures and images for: Intracellular amyloid formation in muscle cells of Aβ-transgenic Caenorhabditis elegans: determinants and physiological role in copper detoxification
Source: Mol Neurodegener. 2009 Jan 6;4:2. doi: 10.1186/1750-1326-4-2 (PMC2632641; doi:10.1186/1750-1326-4-2)

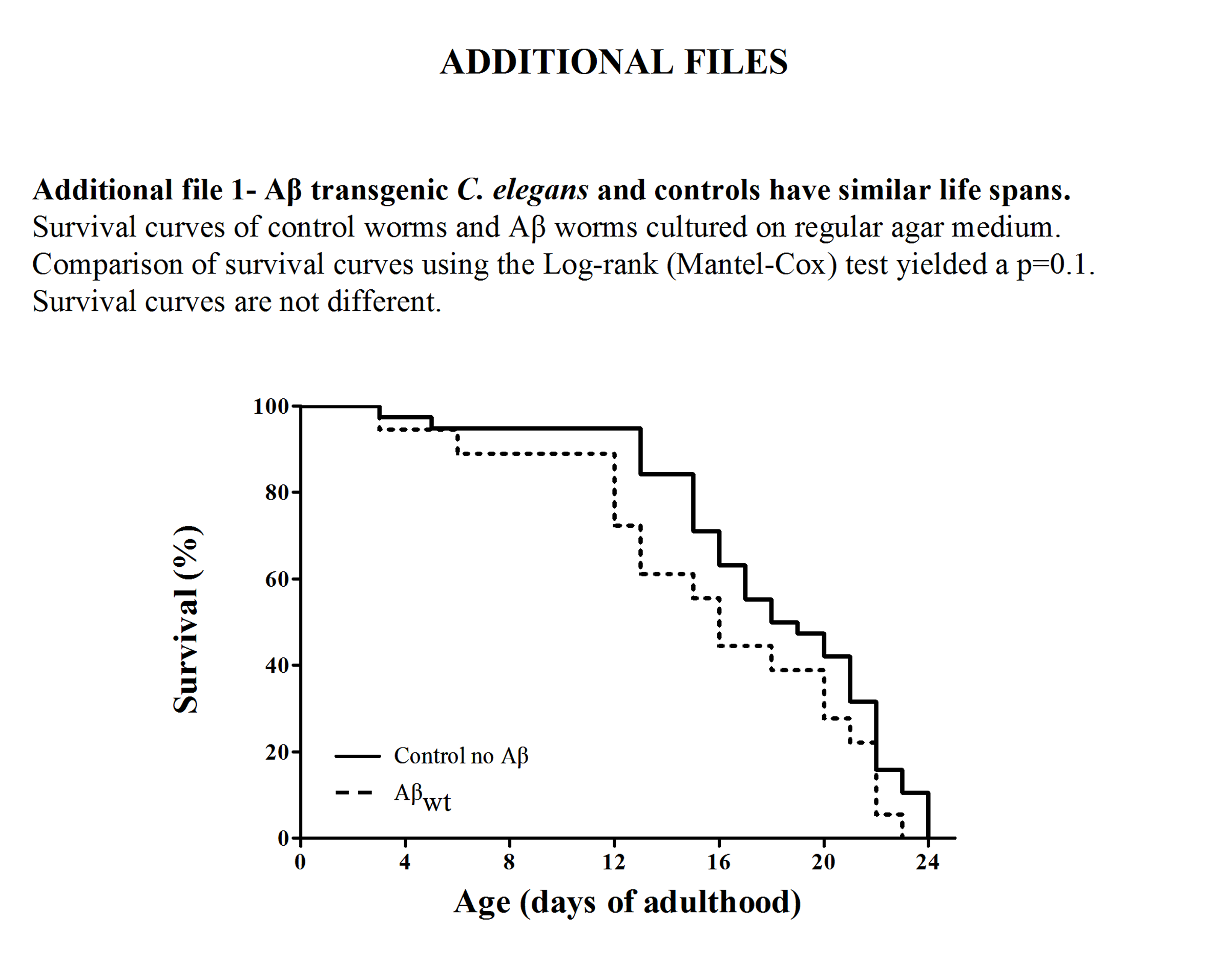

Supplement: Additional file 1 — Aβ transgenic C. elegans and controls have similar life spans. Survival curves of control worms and Aβ worms cultured on regular agar medium. Comparison of survival curves using the Log-rank (Mantel-Cox) test yielded a p = 0.1. Survival curves are not different. [file 1750-1326-4-2-S1.tiff]
